# Cost analysis of breast cancer: a comparison between private and public hospitals in Iran

**DOI:** 10.1186/s12913-021-06136-6

**Published:** 2021-03-11

**Authors:** Abolhasan Afkar, Habib Jalilian, Abolghasem Pourreza, Habibeh Mir, Abdolhosein Emami Sigaroudi, Somayeh Heydari

**Affiliations:** 1 Social Determinants of Health Research Center, School of Health, Guilan University of Medical Sciences, Rasht, Iran; 2 Assistant Professor, Department of Health Services Management, School of Public Health, Ahvaz Jundishapur University of Medical Sciences, Ahvaz, Iran; 3 Social Determinants of Health Research Center, Ahvaz Jundishapour University of Medical Sciences, Ahvaz, Iran; 4 Department of Health Management and Economics, School of Public Health, Tehran University of Medical Sciences, Tehran, Iran; 5 Student Research Committee, School of Management and Medical Informatics, Iran University of Medical Sciences, Tehran, Iran; 6 Cardiovascular Diseases Research Center, Department of Cardiology, School of Medicine, Heshmat Hospital, Guilan University of Medical Sciences, Rasht, Iran; 7 School of Public Health, Guilan University of Medical Science, PO Box: 3391, Rasht, Iran

**Keywords:** Breast cancer, Burden of disease, Direct costs, Indirect costs, Cost of illness

## Abstract

**Backgrounds:**

Breast cancer is the most prevalent cancer among women. Breast cancer imposes a considerable economic burden on the health system. This study aimed to compare the cost of breast cancer among patients who referred to private and public hospitals in Iran (2017).

**Methods:**

This was a prevalence-based cost of illness study. A total of 179 patients were selected from private and public hospitals using the census method. The researcher-constructed checklist was used for data collection. Data were analyzed using SPSS software version 22.

**Results:**

The estimated total mean (SD) direct cost of patients who referred to the private hospital and the public hospital was $10,050 (19,480) and $3960 (6780), respectively. Further, the total mean indirect cost of patients who referred to the private hospital was lower than those referring to the public hospital at $1870 (15 % of total costs) and $22,350 (85 % of total costs), respectively. These differences were statistically significant (*P* < 0.05).

**Conclusions:**

Breast cancer imposes a substantial cost on patients, health insurance organizations and the whole society in Iran. Therefore, the adoption of effective measures for the prevention and early diagnosis of breast cancer is urgently needed.

.

## Highlights

- Hospitalization and outpatient costs in the private hospital were higher compared with the public hospital.
- Indirect costs were higher in the public hospital compared with the private hospital.
- A statistically significant difference was found between supplementary insurance status and total medical direct cost (*P* < 0.05).

## Background

Cancer is a leading cause of death worldwide. The number of cancer cases and deaths is projected to grow rapidly due to population ageing and adopt lifestyle behaviours that increase cancer risk. This is especially important in low- and middle-income countries as they undergo an economic transition [1]. The estimated number of new cases and cancer deaths was 2,088,849 and 626,679, respectively, worldwide in 2018 [2].

Breast cancer is a major public health problem, and 1.7 million new cases are diagnosed per year. It has been shown that almost 60 % of deaths from breast cancer occur in developing countries [3, 4]. In 2018, breast cancer was the most commonly diagnosed cancer in women (24.2 %, i.e. nearly one in 4 of all new cancer cases diagnosed in women worldwide were breast cancer) [5]. In 2018, it was estimated that 627,000 women died from breast cancer, contributing approximately 15 % of all cancer deaths among women [5]. It has been estimated that the incidence of women breast cancer worldwide will reach approximately 3.2 million new cases per year by 2050 [4].

Breast cancer in developing countries represents one-half of all breast cancer cases and 62 % of cancer mortality [6]. In Iran, breast cancer is the fifth leading cause of cancer mortality [7–9]. According to GLOBOCAN database 2018, the number of new cases, deaths and 5-years prevalence from breast cancer for women in Iran was estimated to be 13,776, 3526 and 40,825, respectively [10]. In the last 30 years, the probability of breast cancer incidence for individuals aged 15–79 years in Iran has increased, according to the statistics [11]. According to the statistics, 6160 breast cancer cases are diagnosed in the country each year, and 1063 cases result in death [12]. In 2035 compared to 2012, the number of new cases will be nearly two times greater [13].

Breast cancer imposes a considerable economic burden on societies [14–16]. For example, the total cost of breast cancer was more than three times the total cost of prostate cancer [17]. A study by Figueiredo et al. indicated that public healthcare costs increased between 2004 and 2014, and the correlation between breast cancer and public healthcare costs was positive, mainly influenced by governmental strategies [18]. Breast cancer imposes a significant financial burden on healthcare systems of Iran [19, 20]. Policymakers and health planners are interested in understanding the economic burden of illnesses to assess the optimal allocation of health resources to various diseases and estimate the potential costs and benefits of public health interventions [20].

Cost of Illness (COI) studies indicate the importance of a particular disease and provide a baseline for assessing new interventions [20] and financial losses as a result of illness [21]. The aim of the COI-studies is providing an estimate of how much society spends on a particular disease and identifying different cost components [22]. The COI can be used as a criterion for decision making in allocating limited budgets and resources for governmental health policies in effective control of diseases [21]. A comprehensive economic analysis demands consideration of both direct and indirect costs such as productivity losses as a result of individuals unable to work because of hospitalization or outpatient visits, and also premature death arising from the illness [21].

In future, the cost of cancer care will increase as new sophisticated, expensive treatment modalities are adopted to raise the standard of care [23]. Breast cancer is on the rise in Iran, and since patients are mostly diagnosed at more advanced stages of the disease [24, 25], mortality resulting from breast cancer is high [26]. So, the presentation of accurate data about the economic burden of the disease will allow informed decision making by health care policymakers in Iran about the prevention and treatment of the disease. Therefore, the objective of this study was to compare the cost of breast cancer among patients who referred to private and public hospitals in Iran in 2017.

## Methods

### Database and study population

This was a prevalence-based cost of illness study, which was conducted from the societal perspective using bottom-up approach costing.

The statistical population in this study included all patients with breast cancer. One hundred seventy-nine patients with breast cancer who admitted to the private hospital (*N* = 103) and the public hospital (*N* = 76) in Rasht (a city in the north of Iran) between Aug 2016 and Aug 2017 included in this study.

### Cost assessment

The cost of illness is divided into three general categories: direct costs, indirect costs, and intangible costs. In this study, we mainly focus on the first two cost categories. The direct costs consist of medical costs and non-medical costs. The former includes medical care expenditures for diagnosis, treatment, and rehabilitation, etc., while the latter includes the consumption of non-healthcare resources like transportation, household expenditures, relocating. Indirect costs include lost productivity due to premature deaths and missed workdays and decreased workplace productivity due to morbidity. Finally, intangible costs include the cost of pain and suffering in patients and their families and relatives. In this study, intangible costs were not calculated.

In this study, the economic burden of breast cancer was assessed by calculating direct medical costs, direct nonmedical costs, and indirect costs. Data related to the hospitalization part of direct medical costs were extracted from patients’ records and data related to the outpatient part of direct medical costs, direct nonmedical costs and indirect costs were obtained via an interview with patients and their family members, respectively. The researcher-made checklist was used for data collection. The initial draft of the checklist extracted from two records: (1) “Cost-of-illness studies - a primer” [27] and (2) “Cost-of-illness studies: concepts, scopes, and methods” [28]. Then, to complete the checklist, we interviewed 5 oncologists, 2 researchers who had conducted at least one cost of illness study, 2 professors in the field of Health Economics and 8 breast cancer patients. The checklist consists of demographic variables (age, marital status, monthly income status, educational status, job status, supplemental insurance status, and the type of basic insurance), duration of the disease and treatment type and questions related to costs components incurred by patients during cancer diagnosis, and treatment procedures. In this study, direct medical costs were valued based on the medical tariffs of diagnostic and therapeutic services.

Indirect costs include the monetary value of resources loses due to morbidity and mortality. There are three approaches to estimate indirect costs: the human capital approach (HCA), the friction cost approach (FCA) and the willingness to pay approach (WTP). HCA measures the lost production, in terms of lost earnings, of a patient or caregiver. FCA measures only the production losses during the time it takes to replace a worker, and WTP measures the amount an individual would pay to reduce the probability of illness or mortality. HCA is the most common approach used to calculate the indirect costs of an illness. A criticism of this approach is that certain groups are assigned a higher value than others. A criticism of WTP is that this approach is often difficult to implement in COI studies. For specific diseases, extensive surveys of people’s preferences are needed, which the results rely heavily on the type of question and people’s responses to very specific hypothetical questions. For communicable diseases, surveys may ignore the cost of the disease because of externalities (cost of externalities incurred by disease). The WTP, therefore, is often not feasible for a cost-of-illness study. Proponents of the FCA criticize the HCA for overvaluing the indirect costs, claiming that the productivity losses are often eliminated after a new employee is trained and can replace the former employee. However, the FCA is rarely used because it requires extensive data to estimate losses in the friction period. On the other hand, the estimated cost is strongly influenced by the labour supply situation [27, 29].

In this study, indirect costs were calculated based on the HCA. These costs were estimated by summing two parts: (1) The costs of lost productivity due to patients and their families’ missed workdays and (2) the cost of premature death due to breast cancer. First, in order to estimate the cost of missed workdays per patient, we calculated the average number of missed workdays by patients and their families because of breast cancer and then multiplied by the minimum daily wage rate (310,000 (2017)), in this way we estimated the cost of missed workdays per patient. Also, by having the number and the mean age of premature death and retirement age (60 years old) in Iran, the total number of years lost due to premature death resulting from breast cancer was calculated and multiplied by the number of days of the year and the minimum daily wage rate, in this way the cost of premature death was calculated. Finally, the total cost of lost productivity calculated by summing these two parts.

The equations used for indirect costs calculation are as follows:

1 $$ \mathrm{The}\ \mathrm{cost}\ \mathrm{of}\ \mathrm{missed}\ \mathrm{workdays}=\mathrm{the}\ \mathrm{mean}\ \left(\mathrm{patients}\ \mathrm{missed}\ \mathrm{workdays}+\mathrm{patient}\ \mathrm{family}'\mathrm{s}\ \mathrm{missed}\ \mathrm{workdays}\right)\times \mathrm{minimum}\ \mathrm{daily}\ \mathrm{wage}\ \mathrm{rate} $$

2 $$ \mathrm{C}=\mathrm{the}\ \mathrm{mean}\ \left\{\left(\mathrm{retirement}\ \mathrm{age}-\mathrm{age}\ \mathrm{at}\ \mathrm{premature}\ \mathrm{death}\right)\times \left(\mathrm{the}\ \mathrm{number}\ \mathrm{of}\ \mathrm{patients}\ \mathrm{who}\ \mathrm{died}\div \mathrm{sample}\ \mathrm{size}\right)\right\}\times \left(\mathrm{minimum}\ \mathrm{daily}\ \mathrm{wage}\ \mathrm{rate}\times \mathrm{the}\ \mathrm{number}\ \mathrm{days}\ \mathrm{of}\ \mathrm{the}\ \mathrm{year}\right) $$

To recall bias prevention, patients’ treatment process were followed up every two months for one year.

### Unit costs

All costs in this study were expressed as US Dollars based on the Exchange rate of Central Bank of the Islamic Republic of Iran (US$ 1 = 31,389 Rials (2017)). As the time horizon of the study was one year, costs are not discounted (Table 1).

**Table 1:** Cost categories and sources of applied unit costs

Sector	Service / Goods	Data source	Units	Monetary values (unit costs)
**Surgery costs**	Operating room consumables & equipment, Operating room medication, physician (surgeon) work, anaesthetic	Medical records	Relative value unit/ Current Procedural Terminology	Medical tariffs
**Hoteling costs**	Cost of non-physician human resources, depreciation, repairs and maintenance, food, energy, other goods and services not included in the billing separately	Medical records	Days of hospital stay	Reimbursement schedule
**Diagnostic costs**	Electrocardiography (ECG ), pathology, consulting	Medical records	Quantity	Reimbursement schedule
**Visit Costs**	Visit in hospital	Medical records	Quantity	Reimbursement schedule
**Medication costs**	Medications that are recorded with a separate title in hospital billing codes	Medical records	Quantity	Reimbursement schedule
**Other hospitalization costs**	Intravenous chemotherapy cost, cost of faculty members, mastectomy cost, Forensic medicine cost, hospital cost	Medical records	Quantity	Reimbursement schedule
**Outpatient diagnostic costs**	Screening mammography, diagnostic mammography, ultrasonography, breast MRI	Questionnaire	Quantity	Reimbursement schedule
**Chemotherapy costs**	Manpower cost, drug cost	Questionnaire	Quantity	Reimbursement schedule
**Radiotherapy costs**	Manpower cost, equipment cost	Questionnaire	Quantity	Reimbursement schedule
**Outpatient visit costs**	Physician office visit	Questionnaire	Quantity	Reimbursement schedule
**Other outpatient costs**	Physiotherapy cost, Injection cost, over-the-counter medication price, prescription medication cost, paying extra cost to the surgeon, cost of caregivers, vitamin cost	Questionnaire	Quantity	Reimbursement schedule
**Commuting and food costs**	Costs that patients and caregivers incurred due to commuting to treatment centres	Questionnaire	Quantity	Consumer price
**Accommodation costs**	(costs that patients and caregivers incurred due to residing in hotel or hostel for receiving services in other cities)	Questionnaire	Quantity	Consumer price
**The cost of missed workdays (patient)**	Productivity losses resulting from the disease	Questionnaire	Days	The current average wage in the country
**The cost of missed workdays (family)**	Productivity losses of caregivers for patient care	Questionnaire	Days	The current average wage in the country
**The cost of premature death**	Productivity loss due to premature death	Questionnaire	Years	The current average wage in the country

### Data analysis

Data were analyzed using SPSS software version 22 and excel (2016). Descriptive statistics (mean (SD), frequency, and percent) were used to assess the status of the demographic variables. K-S test (Kolmogorov-Smirnov) was applied to assess the normality of data. Since the *P*-Value for all variables was less than 0.05 (*P* < 0.05), non-parametric tests, including Mann-Withney and Kruskal-Wallis, were used to assess the association between demographic variables and costs. The Spearman correlation coefficient also was used to examine the correlation between age at diagnosis and costs. A multivariate regression model was used to control for confounding factors.

One-way sensitivity analyses were conducted to assess the effects of varying key components of direct medical costs on the total direct medical costs. The variation ranges were established based on the ± 50 % of the index value; index value was set based on the mean total direct medical costs.

## Results

A total of 179 patients with breast cancer were included in the analysis. The majority of patients were covered by the basic insurance (98.9 %), and only 36.3 % of patients were covered by supplemental insurance. Most of the patients (64.2 % ) held a diploma degree and more than half of the patients were non-natives (54.2 %). A statistically significant difference was found between supplemental insurance status and total medical direct cost (*P* < 0.05) Table 2.

The mean(SD) of age at diagnosis, age and age at death was estimated at 45.41 (9.38), 47.98 (10.08) and 49.94 (11.80), respectively. The estimated mean(SD) number of hospital admission and the length of hospital stay of patients who referred to the private hospital was 1.35 (0.50) and 2.71 (2.49), respectively whereas those who referred to the public hospital was higher at 1.48 (085) and 8.63 (1049), respectively. Additionally, 10.7 % of patients who referred to the private hospital and 6.6 % of those referring to the public hospital postponed their treatment process for more than two months due to financial barriers.

**Table 2:** Demographic characteristics and direct medical cost (*N* = 179)

Variable	Modes	N (%)	$Mean (SD)	*P*-Value
**Age**	**< 40 years**	39 (21.8)	9296 (20,500)	0.32
	**40–60 years**	121 (67.6)	6885 (14,600)	
	**> 60years**	19 (10.6)	4930 (8640)	
**Marital status**	**Single**	15 (8.4)	5737 (9910)	0.30
	**Married**	164 (91.6)	7330 (15,980)	
**Education status**	**Illiterate**	40 (22.1)	11,230 (16,100)	0.06
	**Diploma**	115 (64.2)	10,775 (17,390)	
	**Academic education**	24 (13.7)	24,695 (39,880)	
**Supplemental Insurance status**	**Yes**	65 (36.3)	11,427 (23,500)	0.001*
	**No**	114 (63.7)	4795 (7280)	
**Type of Basic insurance**	**Social security insurance**	84 (46.9)	7635 (16,710)	0.79
	**Iranian health insurance**	63 (35.0)	7690 (17,520)	
	**Relief foundation insurance**	19 (10.7)	3595 (4095)	
	**Other basic insurances**	13 (7.3)	6040 (7560)	
**Habitation status**	**Native (patients resident in the city of Rasht)**	82 (45.8)	6857 (16,060)	0.92
	**Non-native (non-Rasht patients)**	97 (54.2)	7480 (15,170)	
**Type of hospital**	**Private hospital**	103 (57.5)	9885 (19,425)	0.001*
	**Public hospital**	76 (42.5)	3620 (6410)	

*P < 0.05 was considered as significant

As shown in Table 3, direct costs in private hospital accounted for 84.04 % of total costs and almost 1.92 times GDP per capita. In contrast, direct costs in public hospital accounted for 17.22 % of the total cost and 75.80 % of GDP per capita.

The direct medical costs of breast cancer patients who referred to the private hospital and the public hospital were $9880 (82.90 % of the total costs and 1.89 times GDP per capita) and $3620 (13.74 % of the total costs and 69.29 % of GDP per capita), respectively. The hospitalization costs and outpatient costs of patients who referred to the private hospital were higher than those referring to the public hospital. The highest component of hospitalization costs of patients who referred to the private hospital was related to surgery cost at $980 (53.73 % of the total hospitalization cost), whereas that of patients who referred to the public hospital was related to hoteling costs at $380 (30.26 % of the total hospitalization cost).

Moreover, medication cost had the lowest rate in breast cancer patients who referred to the private hospital at $30 In contrast, the lowest cost among those referring to the public hospital was related to the diagnostic cost at $100. In summary, outpatient costs were the main component of the direct medical costs for breast cancer patients who referred to the private hospital and the public hospital.

Besides, the total mean nonmedical direct cost of patients who referred to the private hospital and the public hospital was $170 (1.39 % of the total costs) and $340 (1.29 % of the total costs), respectively. The highest component of the direct nonmedical cost of patients who referred to the private hospital and the public hospital was attributed to commuting and food costs at $150and $250, respectively Table 3.

**Table 3:** Mean (SD) and Median (Interquartile Range) of breast cancer costs ($)

Variable	Mean (SD)		Median (Interquartile Range)		*P*-Value
	**Private Hospital**	**Public Hospital**	**Private Hospital**	**Public Hospital**	
**Surgery costs**	980 (610)	195 (335)	950 (225)	0 (350)	0.001*
**Hoteling costs**	360 (420)	380 (550)	210 (250)	170 (400)	0.02*
**Diagnostic costs**	300 (230)	105 (160)	310 (280)	60 (130)	0.001*
**Visit Costs**	35 (95)	140 (230)	0 (5)	70 (185)	0.001*
**medication costs**	30 (80)	265 (530)	15 (20)	40 (340)	0.001*
**Other hospitalization costs**^**a**^	120 (290)	160 (320)	140 (170)	70 (160)	0.01*
**Total hospitalization cost**	1830 (940)	1250 (1550)	1620 (700)	770 (1470)	0.001*
**Outpatient diagnostic costs**	2430 (7440)	425 (940)	0 (1530)	0 (480)	0.12
**Chemotherapy costs**	760 (1200)	195 (540)	15 (1430)	0 (0)	0.001*
**Radiotherapy costs**	550 (1340)	160 (710)	0 (60)	0 (0)	0.001*
**Outpatient visit costs**	460 (1120)	100 (320)	0 (380)	0 (190)	0.01*
**Other outpatient costs**^**b**^	3865 (11,690)	1480 (60)	0 (1810)	0 (730)	0.16
**Total outpatient costs**	8060 (19,370)	2365 (6430)	2350 (7725)	0 (2670)	0.004*
**Total medical direct costs**	9880 (19,425)	3620 (6410)	4250 (8190)	1770 (3730)	0.001*
**Commuting and food costs**	150 (690)	250 (700)	0 (15)	0 (120)	0.37
**Accommodation costs**	10 (125)	920 (570)	0 (0)	0 (0)	0.36
**Total non-medical direct costs**	170 (700)	340 (900)	0 (15)	0 (120)	0.41
**Total direct costs**	10,050 (19,480)	3960 (6780)	4490 (8290)	1935 (4715)	0.001*
**The cost of missed workdays (patient)**	655 (2690)	140 (1220)	0 (0)	0 (0)	0.05*
**The cost of missed workdays (family)**	1215 (3043)	960 (2690)	0 (590)	0 (0)	0.08
**The total of missed workdays**	1870 (4930)	10,100 (3080)	0 (830)	0 (0)	0.03*
**The cost of premature death**	0	21,250 (11,630)	0 (0)	10,640 (19,110)	-
**Total indirect costs**	1870 (4930)	22,350 (18,630)	0 ()	10,640 (19,110)	0.03*
**Total costs**	11,960 (20,530)	22,970 (12,660)	5620 (9230)	11,340 (22,540)	0.001*

*P < 0.05 was considered as significant

^a^intravenous chemotherapy cost, cost of faculty members, mastectomy cost, Forensic medicine cost, hospital cost

^b^Physiotherapy cost, Injection cost, over-the-counter medication price, prescription medication cost, paying extra cost to the surgeon, cost of caregivers, vitamin cost

The total mean indirect cost of patients who referred to the private hospital was $1870, making up 15.69 % of the total cost and 0.35 of GDP per capita, and for those referring to the public hospital was $ 22,350, comprising 84.95 % of the total costs and 4.28 times GDP per capita. This difference was statistically significant (*P* < 0.05). According to our findings, the total missed workdays of patients, and patients’ families who referred to the private hospital were estimated to be 66 and 123 days while those of patients who referred to the public hospital estimated at 14 and 97 days, respectively. Both in the private hospital and the public hospital, the mean cost of lost workdays was considerably higher for family caregivers than for the patients themselves.

A multivariate regression model was used to control for age, education status, marital status, habitation status, type of basic insurance and supplemental insurance status. The results of the regression model are presented in Table 4. Hospitalization costs in the public hospital were 510, significantly lower as compared with the private hospital. Besides, outpatient costs and direct medical costs in the public hospital respectively were 3290 and 5270, lower than the private hospital, but this was not statistically significant.

**Table 4:** Multivariate regression results of direct medical costs

Dependent variable	Independent variables	B(SE)	Beta	*p*-value
**Medical direct costs**	Hospital (reference group = public)	-5270 (6420)	-0.13	0.41
**Hospitalization costs**	Hospital (reference group = public)	-510 (245)	-0.30	0.04*
**Outpatient costs**	Hospital (reference group = public)	-3290 (6020)	-0.09	0.59

*P < 0.05 was considered as significant

The reimbursement rate of basic insurance for patients who referred to the public hospital was higher than those referring to the private hospital (90.68 % VS 37.85 %). Additionally, 26.77 % and 6.39 % of costs were paid by patients who referred to the private hospital and the public hospital, respectively Table 5.

**Table 5:** Mean (SD) of hospitalization costs based on the type of payer ($)

Type of payer	Private Hospital	The total hospitalization cost (%)	Public Hospital	The total hospitalization cost (%)
**Basic insurance**	690 (870)	37.85	1150 (1460)	90.68
**Supplemental insurance**	645 (695)	35.36	0	0
**Subsidy**	0	0	37 (100)	2.92
**Patient**	490 (645)	26.77	80 (90)	6.39

The total mean cost of breast cancer among patients who referred to the public hospital was estimated at $ 22,970, which was equivalent to 4.4 times GDP per capita (Gross Domestic Product per capita = 5219.1 USD (2016), while that of patients who referred to the private hospital was $11,960, which was equivalent to 2.29 times GDP per capita. As shown in Table 6, the major component of the total costs of patients who referred to private hospitals was related to the direct costs at 84.30 % (almost 5.6 times greater than those referring to the public hospital) which was equivalent to 1.92 times GDP per capita. In comparison, the major component of the total costs of patients who referred to the public hospitals was related to the indirect costs at 84.95 % (almost 5.41 times greater than those referring to the private hospital) which was equivalent to 4.28 times GDP per capita.

**Table 6:** Main costs of breast cancer compared with mean total cost and GDP per capita

Costs	Private hospital		Public hospital	
	**Total costs (%)**	**GDP per capita (%)**	**Total costs (%)**	**GDP per capita (%)**
**Medical direct costs**				
**Total hospitalization costs**	15.33	35.02	4.75	23.95
**Total outpatient costs**	67.62	154.48	8.99	45.33
**Total medical direct costs**	82.90	189.39	13.74	69.29
**Non- medical direct costs**				
**Commuting and food costs**	1.29	2.95	0.94	4.74
**Accommodation costs**	0.10	0.23	0.35	1.76
**Total non-medical direct costs**	1.39	3.19	1.29	6.51
**Total direct costs**	84.30	192.59	15.04	75.80
**Indirect costs**				
**The cost of missed workdays (patient)**	5.50	12.56	0.53	2.68
**The cost of missed workdays (patient’s family)**	10.19	23.28	3.64	18.37
**The total cost of missed workdays**	15.69	35.84	4.17	21.06
**The cost of premature death**	0	0	80.78	407.14
**Total indirect costs**	15.69	35.84	84.95	428.20

Variations in sensitivity analysis results are listed in Table 7.

**Table 7:** Model variables: Total direct medical costs

Variables	Base case^a^	Low value	High value
**Medication costs**	131.65	65.82	197.4
**Hoteling costs**	367.15	183.57	550.72
**Radiotherapy costs**	386.19	193.09	579.28
**Visit Costs**	391.14	195.57	586.71
**Chemotherapy costs**	520.68	260.34	781.03
**Surgery costs (Mastectomy)**	648.00	324.00	972.00
**Diagnostic costs**	1791.54	895.77	2687.31

^a^Mean of each variable, Mid line = 4236.37

In our study, midline (= 4236.37) was obtained by summing direct medical costs components. Low and high value was calculated as follows:

(3) Low case = mean of each component – (0.5*base case).

(4) High case = mean of each component + (0.5*base case).

**Fig. 1:**
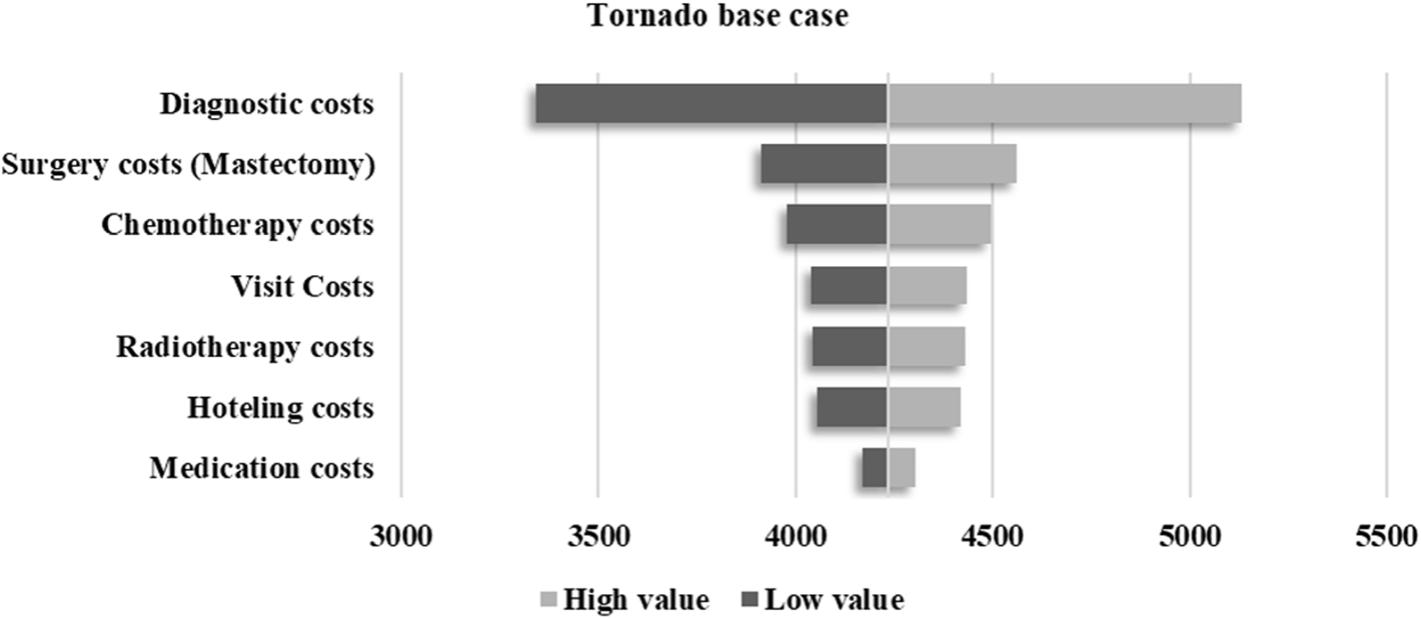
Tornado diagram for one-way sensitivity analysis

Tornado diagrams are presented for the seven main components of direct medical costs (Fig. 1). The results demonstrated that diagnostic, surgery and chemotherapy costs were most important in driving breast cancer costs.

## Discussion

In this study, the estimated mean of age at diagnosis, the age of patients and age at death was 45.41, 47.98 and 49.94 years old, respectively. In our study, the mean age of patients was 47.98 years old, while in Davari et al. (2013), the mean age of patients estimated at 49 years old, in Iran [30]. So it can be concluded that the age of breast cancer onset has decreased in Iran in recent years. The average mortality age of breast cancer is still lower than other cancers, and the economic burden of this disease will rise in the predictable future, according to one study in Japan [21].

In this study, the total mean cost of breast cancer among patients who referred to the public hospital was 1.92 times greater than those referring to the private hospitals (76,630 PPP current international $ VS 41,460 PPP current international $). The results showed that direct costs were the major component of the total costs of patients with breast cancer who referred to the private hospital, whereas the major component of the total cost of those referring to the public hospital was related to the indirect costs.

The indirect cost of patients who referred to the public hospital was 11.94 times as much as than those referring to the private hospital, and this difference was statistically significant. In contrast, the estimated mean medical direct cost of patients who referred to the private hospital was 2.73 times greater than those referring to the public hospital (34264.12 PPP $ VS 12536.16 PPP current international $). In the study of T. A. Dinesh et al., in India, there was a significant difference in the direct cost of care for cancer in private hospitals ($27,425 vs. $21,2320), whereas the indirect cost of care for cancer was significantly higher in government hospitals ($10,340 vs. $6565) [31]. A study by Kounichika et al. in Japan indicated that the mortality costs accounted for 65–70 % of the total cost [21], which the results of these studies are in line with our results.

The difference between direct and indirect costs in patients referred to private and public hospitals may be due to several reasons. Firstly, premature death was the major component of the total indirect cost of breast cancer patients who referred to the public hospital, whereas that did not occur among breast cancer patients in the private hospital. This may be because private hospitals offered better services, resulting in a higher survival rate and a lower mortality rate. Besides, given that the mean age of patients with breast cancer referring to the public hospital (49.776.66 (9.89)) was higher as compared with those referring to the private hospital (46.66 (10.06)), and this difference was statistically significant (*p* < 0.05), the high mortality rate in the public hospital can be because most of the older patients referred to the public hospital. On the other hand, patients with advanced-stage cancer likely referred more to public hospitals for receiving services. Secondly, none of the patients who referred to the public hospital had supplementary insurance, while most of the patients who referred to the private hospital, in addition to basic health insurance, were covered by supplemental insurance. Supplemental insurance has increased patients access to more advanced and expensive treatment services and has made services more inelastic by reducing the patients’ co-payment or have led to increased induced demand. In this study, the correlation between direct medical costs, outpatient costs, chemotherapy costs and age at diagnosis was statistically significant and negative at *P* < 0.05.

Thirdly, tariffs in the private sector are 2–4 times higher than that of the public sector. Therefore, direct medical costs are higher in patients referring to the private sector.

Of note, in Iran, only people who have better socio-economic status and better income level are able to afford supplemental health insurance and refer to the private sector for receiving treatment, which in turn cause they receive more expensive and advanced services. Hence, it may cause the mortality rate among those to refer to the private hospital to be lower and incur lower indirect costs, but due to more treatment services utilization, they are more likely to incur greater direct medical costs than those referring to the public hospital.

Of the direct medical costs, outpatient costs were higher than hospitalization costs in private and public hospitals. The outpatient cost of patients who referred to the private hospital was 3.4 times greater than those referring to the public hospital. The major component of outpatient diagnostic costs of patients who referred to the private and the public hospitals was related to diagnostic costs. Our study results suggest that more attention should be paid to the management of outpatient costs for breast cancer patients in both private and public hospitals. The study by Allaire et al. in the US reported that the outpatient costs caused by breast cancer were equivalent to 94 % of the total cost of breast cancer [32]. In the study of Ekwueme et al., the estimated monthly direct medical costs for breast cancer treatment among younger women enrolled in Medicaid was $5,711 per woman. The estimated monthly cost for outpatient services was $4,058, for inpatient services was $1,003, and for prescription drugs was $539 [33].

Furthermore, the hospitalization costs of patients who referred to the private hospital were 1.46 times greater than those referring to the public hospital. This difference may, in part, be because of the different tariffs or difference in the type of provided services. The most component of total hospitalization cost of patients who referred to the private hospital was related to surgery cost, whilst that of patients who referred to the public hospital was attributable to hoteling cost. In a study by Davari et al., in 2013, in Iran, the main driver of the costs were related to drug therapy [30]. In the study of Omondi Michelle et al., in 2016, patients on chemotherapy alone cost an average of $1364.3; while those treated with surgery cost an average of $1265.6, and those on radiotherapy $1175.1 [34]. A study by Elias et al. showed that the average annual cost of cancer drugs was 6.475$ per patient, which the highest amount of medication costs were related to breast cancer [35].

In the private hospital, the mean of chemotherapy cost for those who had received chemotherapy estimated at $1450 per patient, making up 14.67 % of the total medical costs while in the public hospital was $550 per patient, which accounted for 15.13 % of the total medical costs. Likewise, The mean of radiotherapy cost for those who had received radiotherapy in the private hospital and the public hospital was estimated to be $680 (6.88 % of the total medical costs) and $189 (5.22 % of the total medical costs) per patient, respectively. Moreover, the total direct nonmedical cost of patients who referred to the public hospital was 2.03 times greater than those referring to the private hospital. At both hospitals, commuting costs accounted for the highest component of patients’ total nonmedical direct cost.

The total cost of missed workdays for the patient and the patient’s family, who referred to the private hospital was 1.7 times greater than those referring to the public hospital. Both in the private and the public hospital, the cost of missed workdays of patient’s family members was greater than patients themselves. These costs (opportunity cost) are imposed on patients’ families in real terms but are hidden from policymakers’ view.

In our study, basic insurance played an important role in the reimbursement of direct medical costs and reducing the proportion of out-of-pocket expenses in direct medical costs. The majority of breast cancer costs in public hospitals was paid by basic insurance (90.68 %), 6.39 % of the costs were paid by the patient, and only a small proportion was paid from the targeted subsidy plan by the government (2.92 %). To the contrary, in the private hospitals, %35.36 of costs was reimbursement by supplemental insurance, 37.85 % of costs was reimbursement by basic insurance, and the remaining 26.77 % of costs (6.04 greater than those referring to the public hospitals) was paid by patients. The total out of pocket payments in the private hospital estimated at $3881.23 (approximately 0.38 of total direct costs and 2.83 times higher than in the public hospital), while in the public hospital was $1367.19 (about 0.34 of total direct costs).

It is important to note that although most of the cancer patients in the private sector were covered by supplemental insurance, they paid higher co-payments. Since tariffs in the private sector are 2–4 times higher than that of the public sector, patients referring to private hospitals paid more out of pocket payments despite supplemental insurance. Therefore, these patients are likely to have better socio-economic status and more ability to pay. On the other hand, despite higher costs, these patients may prefer to go to private hospitals because of the shorter waiting time and better service quality.

Since the present study was performed at cross-sectional and prevalence-based method, matching was not conducted between patients referring to the public and the private hospitals in terms of age, income level and disease stage and also the effect of confounding variables was not controlled. Since it is not possible to conclude with any certainty, it is necessary to investigate the cause of this difference in costs and mortality rate between patients referring to the public and the private hospitals in future studies using a perspective and controlled design. In Multivariate regression model after adjusting for confounding variables (e.g., age, education status, marital status, habitation status, type of basic insurance and supplemental insurance status), hospitalization costs in patients referring to private hospitals were significantly higher than those referring to the public hospital. Moreover, our sensitivity analyses showed that diagnostic costs are the key drivers in breast cancer costs. Therefore, it seems the management of diagnostic costs, more than other direct medical costs component, can help to reduce the costs of breast cancer.

### Limitations

This study had several limitations. First, since some patients refused to answer the questions asked of them, the selection bias (sampling bias and attrition) of respondents in reviewing the costs could not be avoided. Second, the indirect costs consisted of only the missed workdays and premature mortality, which would greatly undervalue the indirect economic burden of illness. The lack of data on permanent leaving the job by patients and caregivers during the recovery period could also underestimate the indirect cost estimates. Third, the cost of breastfeeding was not calculated due to the paucity of data. Fourth, intangible economic costs of breast cancer patients and their families, including the pain, sorrow, were not included because they are difficult to convert into a monetary value [36]. Given this was a cross-sectional and prevalence-based study, matching was not conducted between patients referring to the public and the private hospitals in terms of age, income level and disease stage and also the effect of confounding variables were not controlled. An additional limitation is that this study conducted in only two private and public hospitals that can limit the generalization of study findings to all private and public sector.

### Policy implications

Given that the cost of premature death in the private hospital was zero, it is not possible to conclude with certainty whether cancer patients who referred to the public hospital were at the final stage of the disease or benefited from better services or both? If the low mortality rate and low indirect costs in patients referred to the private hospital be attributed to the quantity and quality of services provided to cancer patients referring to the private sector and considering the high share of indirect costs of total costs in patients referred to the public hospital, it is necessary that health policymakers take the necessary measures to improve the quantity and quality of public sector services. Also, despite the insurance coverage, patients suffer a high amount of OOP payment, a substantial and wide-ranging effort is needed to support breast cancer patients. This suggests that insurance policies need to be revised to increase financial support among cancer patients, especially for those who are currently uninsured. It is recommended that the results of this study be used in future studies to evaluate the cost-effectiveness of screening interventions, early detection and preventive interventions, and health policymakers take an appropriate policy to reduce the economic burden of this disease. It is also suggested that future studies should examine whether the higher costs in private hospitals is due to disparities in tariffs of the private and public sector or due to greater quantity and quality services provided in private hospitals.

## Conclusions

Breast cancer imposes a substantial economic burden on patients at private and at public hospitals, healthcare system and society. Indirect costs were considerably higher for breast cancer patients and their caregivers referring to the public hospital, especially in terms of premature mortality than those referring to the private hospital, which can show a significant proportion of the total costs. Because indirect costs do not impose on the health system and health insurance organizations, health policymakers do not pay enough attention to these costs. Therefore, these costs must be addressed at the macro level of economic policymaking. Support strategies also should be adopted for cancer patients and their family members at parliament and government level, and unemployment insurance, improved for cancer patients.

## Data Availability

The data that support the findings of this study are available on reasonable request from the corresponding author S.H. The data are not publicly available due to the data containing information that could compromise participant privacy.

## Abbreviations

COI: Cost of Illness
GDP: Gross Domestic Product
HCA: Human Capital Approach
FCA: Friction Cost Approach
WTP: Willingness to Pay Approach
